# Detection and molecular characterization of hepatitis E virus in food-producing animals in Slovakia

**DOI:** 10.1007/s12223-026-01554-7

**Published:** 2026-07-20

**Authors:** Alica Pavlova, Bozena Kocikova, Michaela Urda Dolinska, Anatolij Kovalenko, Rene Mandelik, Jaroslav Novotny, Anna Jackova

**Affiliations:** 1 Department of Epizootiology, Parasitology and Protection of One Health, University of Veterinary Medicine and Pharmacy in Kosice, Komenskeho 73, Kosice, 041 81 Slovak Republic; 2 Clinic of Swine, University of Veterinary Medicine and Pharmacy in Košice, Komenskeho 73, Kosice, 041 81 Slovak Republic

**Keywords:** Hepatitis E virus, Food-producing animals, Molecular-genetic methods, Slovakia

## Abstract

Hepatitis E virus (HEV) is an emerging public health concern in developed countries, with most infections linked to the foodborne transmission of the zoonotic genotype HEV-3. With increasing number of viral hepatitis E cases in human population in Slovakia, knowledge of the situation in the food-producing animal population is becoming increasingly important. Detection of HEV RNA was performed in 290 samples (collected during period of March 2023 to August 2025) from four different species of food-producing animals – pigs, cows, sheep and goats. The presence of HEV RNA was confirmed by real-time RT-PCR in 5 out of 120 (4.2%) fecal samples from pigs in fattening stage originated from a same farm. Prevalence of HEV RNA was relatively lower than in previous findings, what may reflect limitation in sampling strategy. Phylogenetic analyses of selected ORF1 and ORF2 sequences showed that they clustered within genotype HEV-3f (HEV-3 group 1). Presence of this genotype in Slovak pigs supports possibility of zoonotic transmission, since it is most commonly described in the Slovak human patients with severe course of infection. Despite the fact that HEV is circulating in pigs from Slovakia (*n* = 5/120), we did not detect HEV RNA in cows, sheep or goats (*n* = 0/170). The absence of HEV RNA in food-producing ruminants has been confirmed during our study period in Slovakia, which point to the fact that these species are probably not the main HEV reservoir compared to pigs. Ongoing research will be essential for tracking HEV circulation across reservoirs.

## Introduction

The hepatitis E virus (HEV) is a causative agent of viral hepatitis E, which belongs to the species *Paslahepevirus balayani* within the genus *Paslahepevirus* (Purdy et al. 2022). The HEV genome consists of a single-stranded positive sense RNA containing three open reading frames – ORF1, ORF2, ORF3 (Panda et al. 2007). Phylogenetically, it is divided into eight different genotypes named HEV-1 to HEV-8. Genotypes HEV-1 and HEV-2 are human-restricted, mostly in developing countries and are transmitted through the fecal-oral route (Kamar et al. 2012). Zoonotic genotypes HEV-3 and HEV-4 infect humans and wide range of animals, with most cases associated with foodborne transmission linked to the consumption of raw or undercooked meat and meat products (Tei et al. 2003; Rivero-Juarez et al. 2017). HEV-5 and HEV-6 were described in wild boars, HEV-7 in camels and humans and HEV-8 in Bactrian camels (Lee et al. 2016; Woo et al. 2016; Sridhar et al. 2017).

In Europe, the majority of HEV infections are caused by zoonotic genotype HEV-3 (Schemmerer et al. 2022). Recently many new isolates have been observed, therefore within HEV-3 there are three monophyletic clades, including group 1 (subtypes HEV-3e, 3f and 3g), group 2 (HEV-3a, 3b, 3c, 3h, 3i, 3j, 3k, 3l and 3m) and HEV-3ra (rabbit) (Smith et al. 2020). Pigs are considered to be the main reservoir for this genotype, because of the genetic similarity between HEV strains circulating in pigs and humans, as well as the increasing number of cross-species infection (Ruggeri et al. 2013; Riveiro-Barciela et al. 2015). Albeit less common than in pigs, first cases of HEV infection in food-producing ruminants have also been published (Sarchese et al. 2019; Rahmani et al. 2020), together with first detection of HEV RNA in ovine milk (Dziedzinska et al. 2020). While these infections are typically subclinical in animals, they pose a significant public health risk, because their meat and meat products can cause a serious form of viral hepatitis E in the human population (Rivero-Juarez et al. 2017). Depending on the host immune status asymptomatic, symptomatic acute or even chronic form of infection can be seen (Balaban et al. 2022).

In Slovakia, molecular-genetic studies confirmed only zoonotic genotype HEV-3 with dominance of HEV group 1 in human population and HEV group 2 in animal population (Jackova et al. 2021; Dudasova et al. 2025; Pavlova et al. 2025). Since the zoonotic genotype HEV-3 is circulating in Slovakia, we performed study to determine the HEV prevalence and genetic typing of broader spectrum of food-producing animal species in Slovakia. The consumption of meat and milk products from cows, sheep and goats is popular in Slovakia, but information about HEV presence and genotype distribution in Slovak population of food producing ruminants are missing. Despite being less common reservoirs, we performed first study of HEV RNA presence in fecal samples of cows, sheep and goats.This study may help clarify which food-producing animals can potentially contribute to the rise of the viral hepatitis E in Europe.

## Materials and methods

**Fig. 1:**
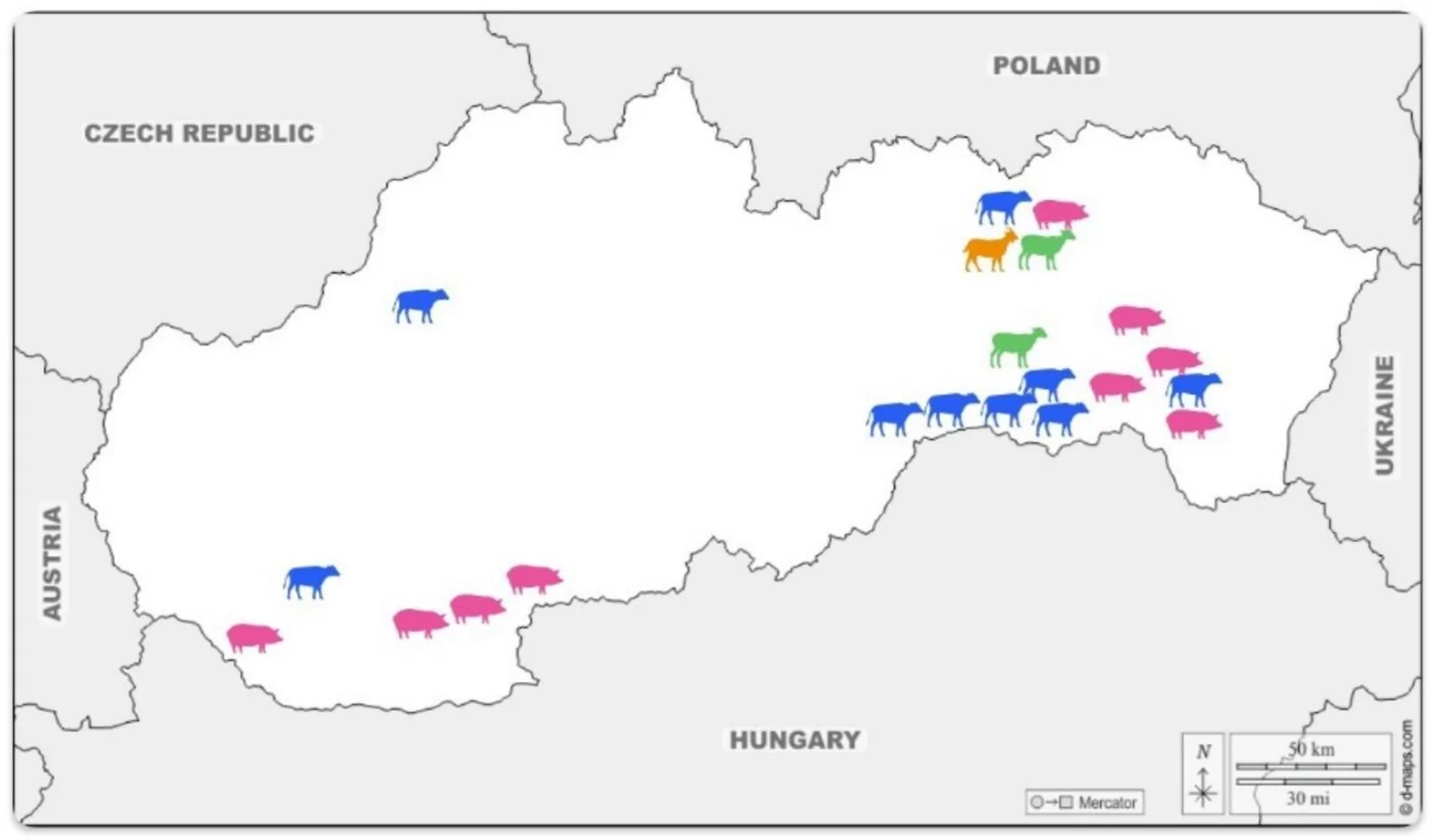
Map of Slovakia indicating the sampling locations with colour marked different animal species. Each animal icon represents a single farm

A total of 290 fecal samples originating from domestic farm animals were collected during the period of March 2023 to August 2025. The samples were collected from animals without clinical signs of infection from 21 different Slovak farms (Fig. 1). The health status of all animals was evaluated by qualified veterinarians on each farm. Samples were collected from 120 pigs (14 days to 8 months old), 100 cows (14 days to 12 months old), 40 sheep (14 days to 12 months old) and 30 goats (14 days to 12 months old). The number of collected samples depended on the size of the farms (10–30 samples per farm). All animal samples in this study were collected with the assistance of their owners and veterinarians and animal welfare was ensured during animal handling. The samples were transported fresh at 4 °C or frozen at −20 °C. After arrival at the laboratory they were immediately processed or stored at −80 °C until the subsequent analysis.

Total RNA was extracted from 140 µL of 10% (w/v) fecal homogenates prepared with 0.9% isotonic saline solution (B. Braun GmbH, Germany) using the QIAamp Viral RNA Mini Kit (QIAGEN GmbH, Germany) on a QIAcube automated platform (QIAGEN GmbH, Germany) according to the manufacturer’s instructions. RNA was eluted in 50 µl of elution buffer with RNA concentration and purity measured by absorbance A260/280. After monitoring of RNA quality it was stored at −80 °C until analysis.

All samples were first screened individually using a broad-spectrum real-time RT-PCR assay targeting ORF3 with primers and a TaqMan probe as described by Jothikumar et al. (2006). Samples with a cycle threshold (Ct) values lower than 40 were considered positive. For every reaction we used positive control – HEV RNA positive sample previously sequenced and verified through BLAST analysis, as negative control we used isotonic saline solution. The house-keeping gene beta actin (ACTB) was applied as an internal control to reduce false-negative detections. The limit of detection (LOD) for the positive control used in the study was determined to be 2.9 log copies/mL. For HEV RNA positive samples a conventional nested PCR assay targeting the 242 bp fragment in ORF1 and 304 bp fragment in ORF2 with primers previously described by Erker et al. (1999) and Meng et al. (1997). The size of amplicons were checked by electrophoresis in a 2% agarose gel after staining with GelRed™ (Biotium, Inc., USA) and visualized by GelDoc Go Gel Imaging System (BioRad, Inc., USA).

For the phylogenetic analysis, PCR products for sequencing of ORF1 fragment (*n* = 3) and ORF2 fragment (*n* = 3) were selected based on DNA quantity and electrophoretic quality, together with positive control. They were purified and sequenced by the Sanger method in a commercial company (Microsynth Seqlab GmbH, Germany). The sequence and phylogenetic analyses were assessed using SeqMan, EditSeq and MegAlign (Lasergene, DNASTAR Inc., USA). Molecular evolution model tests and the phylogenetic trees were created in MEGAX (Kumar et al. 2018). The maximum likelihood phylogenetic trees were built with a Tamura-Nei model with Gamma distribution and Invariable sites (TN93 + G + I) and a bootstrap resampling process (1000 replications) was used to assess node support. Bootstrap values > 70 are indicated at their respective nodes. Analysis of 95% confidence interval (CI) of a proportion was performed by GraphPad Prism 5 for Windows (GraphPad Software Inc., USA).

## Results

A total of 290 fecal samples from pigs, cows, sheep and goats were analysed for the presence of HEV RNA. The results of the real-time RT-PCR indicated that 4.2% (5/120; 95% CI 1.8–9.4%) of pig fecal samples were positive for HEV RNA with Ct values 26.6–34.2. HEV positive samples originated from the same farm in Western Slovakia. All positive pigs were in fattening production stage, the last phase when the animal reaches the market weight. None of the 170 cow, sheep and goat fecal samples were found to be positive (Table 1).

**Table 1:** Detection of HEV RNA by real-time RT-PCR in animal fecal samples

Animal species	Number of samples	Number of + samples (%)	95% CI	Number of farms	Number of samples/farm
Pigs	120	5 (4.2%)	1.8–9.4	9	10–30
Cows	100	0 (0%)	0–3.7	9	10–20
Sheep	40	0 (0%)	0–8.8	2	20
Goats	30	0 (0%)	0–11.4	1	30

**Fig. 2:**
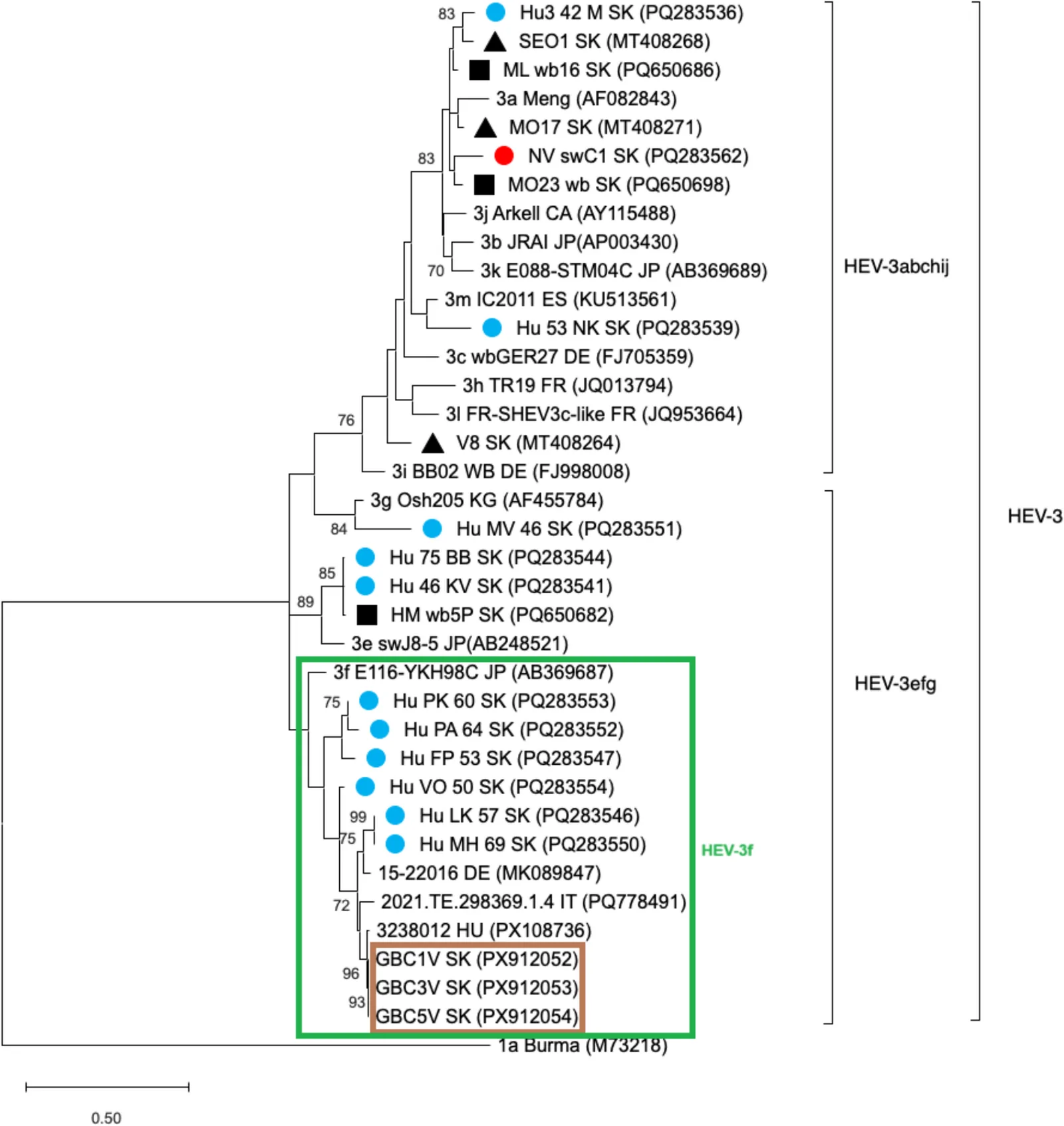
Phylogenetic tree based on the 242 bp nucleotide sequences of the ORF1 fragment of the HEV genome. The HEV sequences from this study are marked with a brown frame, the subtype HEV-3f in a green frame. Sequences from Slovak pig rectal swabs HEV sequences with the black triangles, Slovak pork liver HEV sequence with a red circle, wild boar liver HEV sequences with the black squares and Slovak human patient HEV sequences with the blue circles. All sequences from the GenBank database are marked with sequence name, ISO country code of origin and NCBI GenBank Acc.No. Genotype HEV-1 was used as an outgroup

**Fig. 3:**
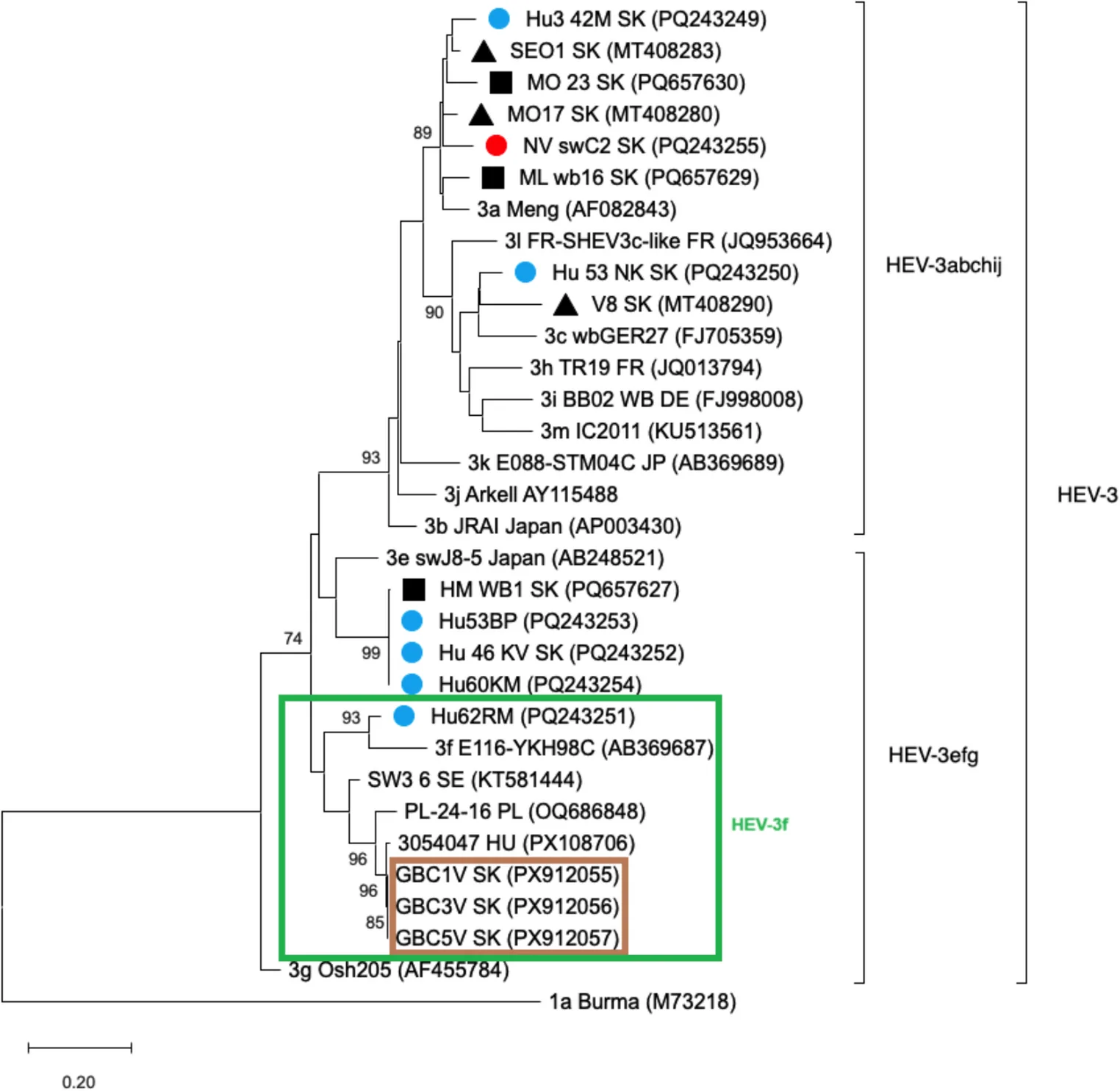
Phylogenetic tree based on the 304 bp nucleotide sequences of the ORF2 fragment of the HEV genome. The HEV sequences from this study are marked with a brown frame, the subtype HEV-3f in a green frame. Sequences from Slovak pig rectal swabs HEV sequences with the black triangles, Slovak pork liver HEV sequence with a red circle, wild boar liver HEV sequences with the black squares and Slovak human patient HEV sequences with the blue circles. All sequences from the GenBank database are marked with sequence name, ISO country code of origin and NCBI GenBank Acc.No. Genotype HEV-1 was used as an outgroup

To determine the HEV genotype in the positive pigs, we further tested three fecal samples that were positive by real-time RT-PCR, analysis of the remaining two samples was not successful due to low quality of PCR products. The obtained sequences were first checked against the NCBI GenBank database using nucleotide BLAST and compared to each other, later with reference sequences described by Smith et al. 2020. Phylogenetic analysis of the ORF1 region from all three partial pig HEV sequences (Fig. 2) revealed that they clustered within genotype HEV-3 within group 1. All sequences were classified as subtype HEV-3f, sharing 100.0% nucleotide identity (nt.id.) with each other and 85.5% nt.id. with the HEV-3f reference strain E116YKH98C (GenBank Acc.No. AB369687). The comparisons with all HEV sequences in GenBank showed that the nucleotide identity of the obtained sequences was closest to human HEV strains from Hungary, Germany and pig HEV strain from Italy (GenBank Acc.Nos. PX108736, PQ778491 and MK089847) with 93.4–98.8% nt.id. Comparison with previously reported Slovak HEV strains showed that they shared 79.3–92.6% nt.id., with the highest nucleotide identity (92.6%) with human HEV strain (GenBank Acc.Nos. PQ283546 and PQ283550). Same three samples were also sequenced in ORF2 region and analysis of the obtained HEV-3 sequences confirmed the results from the ORF1 analysis (Fig. 3). All three sequences were classified same as in the ORF1 region. They belonged to subtype HEV-3f, sharing 100.0% nt.id. with each other and 85.2% with the HEV-3f reference strain E116YKH98C. The comparisons with all HEV sequences in GenBank showed that the nucleotide identity of the obtained sequences was closest to human HEV strain from Hungary and pig HEV strains from Poland and Sweden (GenBank Acc.Nos. PX108706, OQ686848 and KT581444) with 92.8–99.0% nt.id. Comparison with previously reported Slovak HEV strains showed that they shared 79.6–86.5% nt.id., with the highest nt.id. (86.5%) in human HEV strain (GenBank Acc.No. PQ243251). Our study showed that all sequences of the analysed pigs clustered within HEV-3 group 1. Positive control used for every reaction shared 83.5% nt.id. in ORF1 region and 82.9% nt.id. in ORF2 region. The sequence data have been submitted to the GenBank database under Acc.Nos. PX912052 – PX912057.

## Discussion

Shortly after HEV was first identified in the human population, the first detection of HEV in the animal population was described (Balayan et al. 1983; Meng et al. 1997). At the beginning, most cases were travel-associated, but in the last two decades, many autochthonous cases of HEV infection have been reported in developed countries. This study confirmed the circulation of HEV in pigs from Slovakia with 4.2% prevalence. Higher prevalence rates were found in France (8.4%), Spain (11.5%), Italy (14.5%), Hungary (22.7%), the Netherlands (25.6%) and Czech Republic (37.5%) (Reuter et al. 2009; Vasickova et al. 2009; Casas et al. 2011; Jori et al. 2016; Boxman et al. 2022; Ianiro et al. 2023). The low prevalence of HEV RNA in pigs from our study can be influenced by many factors, including the age of tested animals; different sample number, different seasons, sampling method, as well as different laboratory methods used for HEV detection.

Based on the previous studies, piglets can become infected at 8–12 weeks of age, after losing a maternal immunity (Jori et al. 2016; Jackova et al. 2021). In our study, all positive animals were older, in the fattening production stage. All HEV RNA-positive animals originated from the same farm in Western Slovakia and the same age, but considering the different Ct values (26.6–34.2) obtained by real-time RT-PCR, the individual animals were probably at different stages of infection. This finding increases the risk that the infected pigs will reach the slaughterhouse and their meat and products will be used for human consumption. The infection rate can be affected by the occurrence of coinfection just before slaughter and widespread infection on the farms, so the higher proportion of animals shedding virus and the longer shedding period can be present (Salines et al. 2015). Only one pig farm tested positive which may be caused by different farming system, internal and external biosecurity measures and farm management practices.

Previously, HEV-3 was found to be dominant in Slovakia with the majority of animal HEV sequences belonged to HEV-3 group 2 (mostly HEV-3a and HEV-3i) and with human HEV sequences belonged to HEV-3 group 1 (mostly HEV-3f and HEV-3e) (Jackova et al. 2021; Dudasova et al. 2025; Pavlova et al. 2025). Pig HEV sequences obtained in our study belonged to HEV-3 group 1, specifically to subtype HEV-3f. The subtype HEV-3f is the most frequently detected in human patients from Slovakia (Pavlova et al. 2025). Therefore its current detection in the Slovak pig population indicates important epidemiological significance and points to the possibility that Slovak meat and meat products may be a source of infection for their consumers. Phylogenetic analyses confirmed that the highest nucleotide identity of the obtained sequences in ORF1 region was closest to human HEV strains from Hungary, Germany and pig HEV strain from Italy (GenBank Acc.Nos. PX108736, PQ778491 and MK089847) (Fig. 2). Sequence comparison in the ORF2 region revealed that the highest nt.id. was with human HEV strain from Hungary and pig HEV strains from Poland and Sweden (GenBank Acc.Nos. PX108706, OQ686848 and KT581444). The subtype distribution varies in different European countries: pig strains originated from Austria, Bulgaria, Czech Republic, Poland, Germany, Italy, Netherlands, Bulgaria and the United Kingdom showed subtypes HEV-3e, 3f, and 3c were most common (De Sabato et al. 2023). The most frequent subtypes in European human population are HEV-3f, 3e and 3c (Schemmerer et al. 2022; De Sabato et al. 2023). In Slovakia, this is the first detection of HEV-3f genotype in pig population. Obtained pig HEV sequences in both regions were closest to Slovak human strains. These findings and comparisons confirmed genetic similarity between HEV strains circulating in pigs and humans in Europe.

To the best of our knowledge, this is the first study to assess HEV circulation in food-producing ruminants from Slovakia. In the present study, none of the 170 fecal samples collected between 2023 and 2025 were positive for HEV RNA. The total absence of HEV RNA has been also described in cows from several European countries: Hungary, Croatia, Germany, Belgium and Portugal (Reuter et al. 2009; Prpić et al. 2015; Baechlein and Becher 2017; Vercouter et al. 2018; Santos-Silva et al. 2025). Circulation of HEV in cows has been reported in China, Korea and Brazil (Yu et al. 2009; Yan et al. 2016; Go et al. 2019; Bastos et al. 2022). In Europe, only a study from Romania has reported HEV in cows, but the study did not determine the HEV genotype (Rahmani et al. 2020). Recently, the first information of zoonotic HEV-3 presence in sheep and goats have been reported in Italy and Czech Republic (Sarchese et al. 2019; Palombieri et al. 2020; Dziedzinska et al. 2020). We cannot definitively conclude that the virus was not circulating in Slovakia, as would be suggested by the absence of HEV RNA, but the HEV circulation could likely be very limited in cows, sheep and goats, each representing a possible spill-over host. Absence of HEV RNA in food-producing ruminants in our study can be influenced by many factors: different sampling strategy; number of samples, farms and region; sampling period; as well as laboratory methods used for HEV detection. Future research should expand the number of examined samples from more farms, from different regions, so that we can determine whether HEV circulation in food-producing animals is truly absent or simply occurs at very low levels.

The present study provides new insights into actual status of HEV occurrence in domestic food-producing ruminants in Slovakia.

## Conclusion

The zoonotic nature of HEV transmission in European countries has become an emerging issue, especially in immunocompromised patients. This study confirmed the presence of zoonotic HEV-3 in food-producing animals from Slovakia. HEV RNA was detected in pigs in fattening production stage, which suggesting the risk that infected pigs will reach the slaughterhouse and their meat and products will be used for human consumption. The HEV strains obtained in this study has confirmed a high similarity between animal and human HEV sequences. None of food-producing ruminants were positive for HEV RNA during study period. These species probably do not represent the risk for public health in Slovakia, but future research should consider conducting more comprehensive epidemiological surveys.

## Data Availability

The nucleotide sequences generated in this study have been deposited in the NCBI GenBank database under accession numbers PX912052–PX912057.
